# Comparative effectiveness of in-person vs. remote delivery of the Common Elements Treatment Approach for addressing mental and behavioral health problems among adolescents and young adults in Zambia: protocol of a three-arm randomized controlled trial

**DOI:** 10.1186/s13063-022-06319-4

**Published:** 2022-05-19

**Authors:** Caleb J. Figge, Jeremy C. Kane, Stephanie Skavenski, Emily Haroz, Mwamba Mwenge, Saphira Mulemba, Luke R. Aldridge, Michael J. Vinikoor, Anjali Sharma, Sachi Inoue, Ravi Paul, Francis Simenda, Kristina Metz, Carolyn Bolton, Christopher Kemp, Samuel Bosomprah, Izukanji Sikazwe, Laura K. Murray

**Affiliations:** 1 Johns Hopkins Bloomberg School of Public Health, Department of Mental Health, 615 N. Wolfe Street, Baltimore, MD 21205 USA; 2 Department of Epidemiology, Columbia University Mailman School of Public Health, 722 W 168th St., New York City, NY 10032 USA; 3 The Centre for Infectious Disease Research (CIDRZ) Zambia, Plot 34620, Lusaka, Zambia; 4 Department of Medicine, University of Alabama at Birmingham, 845 19th Street South, Birmingham, AL 35294 USA; 5 Department of Medicine, University of Zambia, PO Box 50110, Lusaka, Zambia; 6 Department of Epidemiology, Harvard T.H. Chan School of Public Health, 655 Huntington Ave, Boston, MA 02115 USA; 7 Department of Psychiatry, University of Zambia, PO Box 50110, Lusaka, Zambia; 8 Ministry of Health Zambia, Haille Selassie Avenue, Ndeke House, P.O. Box 30205, Lusaka, Zambia; 9 Department of Global Health, Hans Rosling Center, University of Washington School of Public Health, 3980 15th Ave. NE, Seattle, WA 98105 USA

**Keywords:** Global mental health, Adolescents, Randomized controlled trial, Telehealth, Zambia, Implementation science

## Abstract

**Background:**

In low- and middle-income countries (LMIC), there is a substantial gap in the treatment of mental and behavioral health problems, which is particularly detrimental to adolescents and young adults (AYA). The Common Elements Treatment Approach (CETA) is an evidence-based, flexible, transdiagnostic intervention delivered by lay counselors to address comorbid mental and behavioral health conditions, though its effectiveness has not yet been tested among AYA. This paper describes the protocol for a randomized controlled trial that will test the effectiveness of traditional in-person delivered CETA and a telehealth-adapted version of CETA (T-CETA) in reducing mental and behavioral health problems among AYA in Zambia. Non-inferiority of T-CETA will also be assessed.

**Methods:**

This study is a hybrid type 1 three-arm randomized trial to be conducted in Lusaka, Zambia. Following an apprenticeship model, experienced non-professional counselors in Zambia will be trained as CETA trainers using a remote, technology-delivered training method. The new CETA trainers will subsequently facilitate technology-delivered trainings for a new cohort of counselors recruited from community-based partner organizations throughout Lusaka. AYA with mental and behavioral health problems seeking services at these same organizations will then be identified and randomized to (1) in-person CETA delivery, (2) telehealth-delivered CETA (T-CETA), or (3) treatment as usual (TAU). In the superiority design, CETA and T-CETA will be compared to TAU, and using a non-inferiority design, T-CETA will be compared to CETA, which is already evidence-based in other populations. At baseline, post-treatment (approximately 3–4 months post-baseline), and 6 months post-treatment (approximately 9 months post-baseline), we will assess the primary outcomes such as client trauma symptoms, internalizing symptoms, and externalizing behaviors and secondary outcomes such as client substance use, aggression, violence, and health utility. CETA trainer and counselor competency and cost-effectiveness will also be measured as secondary outcomes. Mixed methods interviews will be conducted with trainers, counselors, and AYA participants to explore the feasibility, acceptability, and sustainability of technology-delivered training and T-CETA provision in the Zambian context.

**Discussion:**

Adolescents and young adults in LMIC are a priority population for the treatment of mental and behavioral health problems. Technology-delivered approaches to training and intervention delivery can expand the reach of evidence-based interventions. If found effective, CETA and T-CETA would help address a major barrier to the scale-up and sustainability of mental and behavioral treatments among AYA in LMIC.

**Trial registration:**

ClinicalTrials.govNCT03458039. Prospectively registered on May 10, 2021

## Background

The quality and availability of mental and behavioral health care in low- and middle-income countries (LMIC) are significantly deficient [1–3]. The high need and inadequate resourcing for mental health results in a substantial treatment gap [1, 2, 4] that directly contributes to the ongoing violation of human rights, abuse and neglect, reduced adherence to medical regimens, long-term disability, ill-health, lower economic productivity, and increased mortality [5–9].

Adolescents and young adults (AYA) in LMIC are in critical need of access to mental and behavioral healthcare. Mental and behavioral health problems account for up to 30% of disability-adjusted life years before the age of 30 [10]. AYA are disproportionately affected by the HIV epidemic, accounting for 30% of all new HIV infections [11]. In Zambia, the location of the current study, AYA are at high risk of HIV infection, poverty, experienced violence (including sexual and gender-based violence), and unemployment [12, 13]. The combination of past and continuing stressors can cause stress-related problems including maladaptive behaviors, emotional and/or behavioral dysregulation, cognitive challenges, substance use/abuse, aggression, risky sexual behavior, and difficulties with functioning [1, 14–20], which in turn increase the risk for a range of poor outcomes, including HIV/AIDS risk, substance abuse, and lower economic productivity [8, 21–23].

The body of research and policy supporting the effectiveness of mental health treatments is growing rapidly [24–28]. Research shows that certain mental health treatments are effective, acceptable, feasible, and can be implemented in LMIC with positive clinical outcomes using an apprenticeship model and a task-sharing approach where lay providers with limited formal mental health training function as counselors [24, 25, 28–31]. Despite this evidence, the wide-scale update and sustainability of non-professional delivered interventions in LMIC are virtually non-existent.

The Common Elements Treatment Approach (CETA) is an intervention that is flexible, transdiagnostic, and delivered by lay counselors or non-professionals in LMIC to address comorbid mental and behavioral health problems [29]. CETA is grounded in cognitive behavioral therapy elements common to evidence-based treatments (EBTs) for trauma, behavioral problems, anxiety, and depression [32]. This approach allows a counselor to decide on which element(s), order, and dose are most appropriate for each client based on presentation. Randomized controlled trials (RCT) have demonstrated the effectiveness of CETA for a range of mental health, substance use, and behavioral issues among adults in a variety of low-resource settings [33–38]. In a non-randomized study in Ethiopia, CETA improved mental and behavioral health problems improved among youth [39]; however, CETA has not yet been evaluated among adolescents in a fully powered randomized trial.

Trainings for EBTs, such as CETA, are typically provided by an “expert” who usually is a Ph.D. level expatriate with extensive experience and EBT purveyor-approved. After the trainings, the “expert” continues EBT supervision and coaching to ensure skill transfer and fidelity [40]. Multiple studies completed on EBTs in LMIC have demonstrated the use of expert-delivered training with the apprenticeship model is feasible and effective [24, 25, 28, 31]. However, scale-up and sustainability efforts of EBTs are nearly impossible when utilizing an expert-delivered training approach since experts are scarce, costly, have limited time, usually require a translator, and often need to travel long distances for on-site trainings, something that was particularly problematic during the COVID-19 pandemic.

A train the trainer (TTT) strategy is a specific form of capacity building designed to provide trainees with the necessary knowledge and skills to become trainers themselves—and, arguably, sustain a workforce over time. The TTT model has been effectively used in a diverse range of disciplines (e.g., education, health care) [41–44] and has involved multiple types of trainees (e.g., direct care counselors, parents, or teachers) depending on the complexity of skills being taught [45, 46]. However, there is limited research and few rigorous studies on whether TTT efforts achieve their desired level of change, particularly with mental health EBT [47]. Research also suggests that technology-based training approaches (i.e., video and audio-based instruction) may have the potential to result in comparable learning to face-to-face instruction across LMIC settings [48, 49]. Thus, applying technology-based training approaches to TTT may increase the scalability of EBTs [50, 51].

In addition to technology-based training of trainers, technology-based delivery of health interventions has been identified as a potential strategy to expand mental healthcare access in LMIC settings [52]. However, most of the evidence showing the efficacy of technology-based mental health delivery has been conducted in high-income countries (HIC) with only discussion of its implications for implementation in LMIC [53, 54]. The majority of studies in this area have focused on videoconferencing, with documented high satisfaction and acceptability of tele-mental health delivery for children and AYA that are underserved or located in rural areas [55–58]. During the COVID-19 pandemic, some mental health practitioners in upper-income countries transitioned almost universally to teletherapy. In various LMIC settings, tele-mental health delivery has been shown to be feasible [59, 60] and similar to HIC contexts and satisfactory comparable to in-person visits [61]. However, there is limited evidence on treatment effectiveness and symptom reduction with technology-based delivery relative to in-person implementation in limited-resource settings [61].

Tele-mental health delivery methods reduce common barriers to treatment access and adherence, including time, financial, and transportation demands, among hard-to-reach populations, particularly during public health crises. Given the rapid increase to access to mobile technology in recent years, tele-mental health delivery has become a viable option. In January 2021, there were over 19 million mobile phone subscriptions in Zambia, an equivalent of about 104% of the population [62]. In addition, previous technology-based healthcare delivery systems have found high user acceptance and increased adoption rates of mobile technology that is low-cost, readily available, culturally sensitive, and easy to operate [63]. Exploring technology options and models for treatment *delivery* is critical for providing and scaling up care in hard-to-reach populations and reducing the treatment gap in LMIC settings [64, 65]. Together, tech-delivered training and telehealth delivery can reduce the need for in-person logistic demands across the training and treatment cascade at the trainer, counselor, and client levels, increasing the feasibility, sustainability, and cost-effectiveness of evidence-based mental health care provision in LMIC settings.

Given the substantial mental and behavioral health treatment gap for AYA in LMIC and the potential for technology to improve the delivery and sustainability of EBTs, research into the effectiveness and feasibility of technology-based approaches to training and intervention delivery is warranted. In this paper, we describe the protocol for a randomized controlled trial to test the comparative effectiveness of traditional in-person CETA and telehealth-delivered CETA (T-CETA) featuring lay counselors trained by trainers through a technology-based TTT approach in reducing mental and behavioral health problems among AYA in an urban setting of Zambia.

## Methods

### Study setting

All study procedures will take place in Lusaka, Zambia. Participants will be recruited from community-based organizations that provide health, education, and social services to AYA in Lusaka. Organizations with access to AYA populations and with existing counseling/mental health services were selected, as they will serve as prime CETA and T-CETA service delivery points, should the study demonstrate feasibility and effectiveness in this population. Selected organizations include a combination of local and international non-governmental organizations (NGOs) and government health clinics. Heterogeneity in implementing sites is desirable to generate robust clinical and implementation evidence.

### Overview of study design

**Fig. 1:**
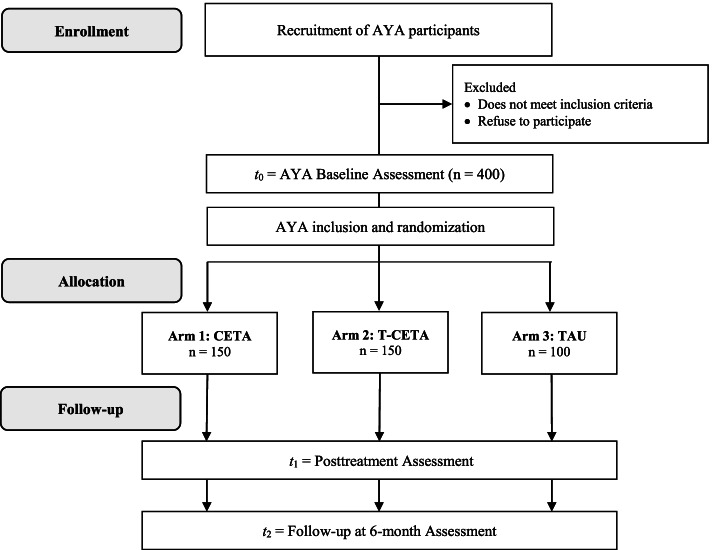
Spirit flow diagram of the trial schedule

This study will use a hybrid type 1 three-arm parallel-group randomized controlled design [66] to compare the effectiveness of CETA delivered either in-person or via telephone (T-CETA), compared with a treatment as usual (TAU) control group. CETA trainers (up to *N* = 6) will be identified from an existing cadre of Zambian counselors and will be trained to be CETA trainers via a technology platform. They will be trained to facilitate technology-delivered CETA trainings to new prospective counselors (up to *N* = 50) recruited from our partnering organizations, who will be trained to deliver both in-person CETA and T-CETA. We will recruit and randomize AYA clients (*N* = 400) with mental and behavioral health problems from our partnering organizations to one of the following three conditions: (a) CETA, (b) T-CETA, or (c) TAU. We will evaluate mental and behavioral health outcomes among AYA at baseline, post-treatment (approximately 3–4 months post-baseline), and 6 months post-treatment (approximately 9 months post-baseline) (Fig. 1). Data will also be collected on CETA trainer and counselor competency, fidelity, and knowledge, and implementation constructs (e.g., acceptability, feasibility, cost) associated with training and intervention delivery.

### Participants

AYA participants will be between the ages of 15–29 with mental and behavioral health problems as measured by validated screening tools (see the “Screening and baseline assessment” section). Trainer and counselor participants will be lay providers interested in mental health training. Full eligibility criteria are summarized in Table 1.

**Table 1:** Participant eligibility and screening

**AYA**	
1. 15–29 years of age	
2. Attend or be referred to the study site	
3. Live in the area served by a study site (i.e., not staying temporarily)	
4. Ability to speak one of the study languages (English, Bemba, or Nyanja)	
5. Screening: present with one or more common mental/behavioral health problems based on validated screening tools included in the audio computer-assisted self-interviewing (ACASI) system. Specifically, the following screening tools and cutoff values:	
a. Youth Self Report Internalizing Scale (≥ 14)	
b. Youth Self Report Externalizing Scale (≥ 8)	
c. Child PTSD Symptom Scale (≥ 12)	
6. Exclusion:	
a. Currently on unstable psychiatric drug regimen (e.g., altered in past 2 months)	
b. Suicide attempt or active and severe self-harm in the past month	
c. Psychotic disorder or severe mental illness	
**Trainers and counselors**	
1. 18 years of age or older	
2. Interest in providing CETA	
3. Time/availability to participate in the study	
4. Minimal education level is comparable to a high school education	
5. Ability to speak English fluently and speak at least 1 local language (Nyanja or Bemba)	
6. Completion of an in-person interview with study team investigators demonstrating strong communication skills	
7. Planning to stay in the study area (Lusaka) to provide treatment to clients and/or training to new counselors	
Trainers: in addition to all of the above:	
8. Interest in teaching CETA	
9. Completion of the CETA training	
10. Completion of a minimum of 3 CETA cases under supervision	

### Recruitment

For the recruitment of AYA clients, the research team will conduct half-day training sessions explaining CETA, and for whom and what problems it is appropriate, to all staff at collaborating partner organizations. In addition, a separate 2-h CETA information meeting will be conducted for home-based care workers that help link individuals to services. Recruitment by home-based care workers connected to partner sites is a process used previously by the research group to help identify, connect, and assure that those in need get services and mirrors how the program could recruit clients in a real-world setting [67, 68]. Initial contact will be made by the partner organization staff or home-based care workers who will explain the study with a recruitment script to AYA, and their primary caregiver for those under age 18, with whom they have previously or currently worked. Snowball recruitment methods may also be used to identify additional AYA participants. Recruitment will be conducted in private locations such as a private room in a community location (e.g., church, school) or a private room within the partner organization’s facilities. AYA that express interest in receiving more information on the study will be connected to the research team.

Trainer- and counselor-level participants will be recruited from our collaborating partners and through contacts of local stakeholders like the Ministry of Health to increase the likelihood that the provision of CETA continues after the end of the study period. Our collaborating partners and contacts will recommend the current staff who they think would be appropriate CETA counselors and have time to provide this service. Potential counselors will also need to submit a resume to the study team, a letter expressing their motivation to be a counselor, and a letter of support from their organization. The study staff will interview the applicants and ensure they meet the inclusion criteria. The final agreement will be by consensus of the study team and collaborating partners.

### Informed consent

Consent activities with all participants involve discussing relevant study details including the purpose, treatment details, privacy, and risks and benefits. Informed consent will be obtained from AYA clients (and caregivers, if applicable) in English, Bemba, or Nyanja by trained research assistants. Consent is obtained for participation in all study activities at the screening phase. AYA will be informed that study participation is voluntary and will not impact any ongoing or future services they may receive. Research assistants will be trained in human subjects and responsible for the conduct of research and receive appropriate certification. For clients under age 18, research assistants will also obtain informed consent and permission from the AYA’s primary caregiver to obtain assent from the AYA participant. Informed consent forms are available from the corresponding author on request.

### Screening and baseline assessment

The screening portion of the baseline assessment consists of demographic information, internalizing and externalizing symptoms, and post-traumatic stress. Screening instruments will be administered using the audio computer-assisted self-interviewing (ACASI) system. Participants can navigate through the questionnaires themselves using a laptop computer while listening to the questions through headphones and seeing the question and response options on the screen. A research assistant will be positioned nearby in case the participant has any difficulty. We have used ACASI extensively with AYA populations in Zambia and found it to be an acceptable and feasible approach for measuring mental and behavioral health outcomes [69]. All measures included in the ACASI have previously been used by our team with similar study populations and are available in English, Bemba, and Nyanja [35, 68–72].

#### Broadband Mental Health Functioning (Youth Self Report (YSR) [72];)

The YSR is a 112-item measure of broadband mental health symptoms on a 3-point Likert scale (*0 = not true, 1 = somewhat true, 2 = very true or often true*). The measure produces 8 subscales and two broad internalizing and externalizing symptom scores. Eligibility cutoffs of ≥ 14 and ≥ 8 will be used for the internalizing and externalizing subscale scores, respectively, based on our previous validity study [73].

#### Post-traumatic stress symptoms (Child PTSD Symptom Scale (CPSS) [74];)

The CPSS is a 17-item scale that corresponds to the DSM-5 criteria for PTSD on a 4-point Likert scale (*0 = not at all or only one time, 1 = once a week or less/once in a while, 2 = 2 to 4 times a week/half the time, 3 = 5 or more times a week/almost always*). The items produce a total Symptom Severity Scale. Based on our previous validity study in Zambia, an eligibility cutoff of ≥12 will be used [73].

Participants who do not meet the eligibility cutoffs on screening measures will exit the study and be provided resources for mental health and other social services in their area. Eligible AYA participants will continue to complete the second portion of the baseline assessment via the ACASI system, which includes measures of substance use, aggression/violence, functioning, and COVID-19 vaccine hesitancy (see the “Outcomes” section).

### Randomization and blinding

Each participant will be assigned a unique ID number by the research assistant during the screening/baseline assessment. Randomization will occur at the individual AYA level and be stratified by site. Following the determination of eligibility and completion of the baseline assessment, the research assistant will communicate the ID number to a study staff based at research headquarters who will not interact with study participants. This study staff will maintain a randomization sequence that will be developed a priori by a US-based research staff. This list will not be available or viewable to data collectors in Zambia. The list will have a sequence of treatment assignments (CETA or T-CETA or TAU) in random order, stratified by site, produced via a random number generator in Microsoft Excel. Randomization will be imbalanced (1.5 CETA: 1.5 T-CETA: 1 TAU) due to the different sample size requirements in the TAU control arm (see the “Data analysis” section). Eligible AYA participants will be assigned a condition based on the next available slot on the randomization list and inform the field staff of the assignment. At this point, the field staff will not be blinded to condition assignment for participants they assess. Eligible AYA clients will be informed immediately of the result of the randomization and appropriate next steps: CETA and T-CETA participants will be told that a counselor will be contacting them within 48 h to set up the first session; TAU participants will be told that the study team will contact them in approximately 3 months to schedule their first post-assessment. The use of ACASI will result in the outcome assessments being blinded; data analysts will also be blind to the intervention status when conducting analyses. Due to the nature of the intervention, neither counselors nor AYA clients will be blinded.

### Intervention arms

#### CETA

The Common Elements Treatment Approach (CETA) is a transdiagnostic, multi-problem intervention designed to address adult and youth trauma, depression, anxiety, safety, and substance use [75]. It comprised a small set of common elements found to be efficacious and prevalent across a range of EBTs to treat common mental health problems. CETA was designed to be flexible in the elements utilized, their order, and their dose (number of sessions) to allow counselors to address heterogeneity, comorbidity, and symptom fluctuations in and across clients. Treatment typically consists of 6 to 12 weekly, approximately 60-min sessions delivered by lay workers.

#### T-CETA

For the adaptation of the CETA manual for telephone delivery, we reviewed evidence-based telehealth strategies and recommendations, telehealth ethical and legal guidelines, and clinical recommendations from telehealth providers. In addition, local trainers-in-training in multiple contexts reviewed telehealth modifications and provided input that was incorporated into the final T-CETA manual used in this study. No changes were made to the structure, duration, and dose of CETA sessions, treatment components, or measurement-based clinical decision-making processes. Telehealth modifications, additions, and strategies were incorporated throughout the manual in delineated “telehealth boxes.” This way, the original manual was maintained outside of the telehealth boxes, allowing for clear identification and training of telehealth modifications for both new and existing CETA counselors. A pilot phase was conducted in Zambia to explore the feasibility and acceptability of T-CETA and to obtain feedback for modifying the telehealth additions or implementation strategies. Qualitative results highlighted the barriers to T-CETA treatment (i.e., poor connection; client phones off or not having phones; lack of private spaces) and solutions to these barriers (i.e., using community staff’s phone for sessions; obtaining multiple contacts for participants; home visits). For those with access to a telephone, T-CETA was deemed by participants as acceptable and effective.

##### Treatment as usual control

Participants randomized to the TAU condition will be encouraged to continue engaging with support services normally offered in their community, such as those provided by the partnering organization, and the types and utilization of these services will be tracked. Counselors will check in with TAU participants monthly to assess safety; any participant with a safety risk will be contacted by a counselor to complete a safety plan and be monitored. All TAU participants will receive a full course of CETA, if desired, after completion of the active study aims. For all study arms, a dedicated adherence team will be identified at each partner site and overseen by the project manager. Adherence strategies will include regular telephone check-ins, coordination with other site providers, and, if needed, home visits. Following the participation in the trial, participants will be provided a list of available resources in the community and the option to reengage with CETA providers in the area, if needed.

##### CETA training

CETA training and supervision activities in this study build on the existing apprenticeship model for lay providers to become mental health counselors [76]. In this model, the trainer initially assumes responsibility for teaching core skills and guiding the prospective counselor. After the initial training, supervisors, who may be identified beforehand or from the cohort of new counselors, are responsible for acting as the link between counselors and trainers. Before counselors begin delivering the intervention to clients, supervisors run practice groups with prospective counselors, which are then followed by supervision groups. Practice groups involve role-plays of a particular intervention component or skill, while supervision groups involve each prospective counselor going through one or two pilot cases with a supervisor who has previously piloted the case themselves with a trainer. When supervisors are providing guidance during practice and supervision groups to counselors, supervisors also engage in weekly calls with a trainer to receive consultation and support. At every level, each individual grows in their role over time and develops the necessary skills so that those who deliver coaching and support eventually provide minimal guidance. For this study, trainers-in-training (*N* = up to 6) will learn how to train new counselors and supervisors via the technology platform.

The technology-based TTT developed for this study consists of 10 days of trainers-in-training reviewing CETA components on the tech platform, expert CETA trainers modeling training components live via video chat and live and pre-recorded video observation of trainers-in-training role-plays by expert CETA trainers. Role-plays by trainers-in-training will be rated using a structured rating system, with feedback provided during live video sessions.

Following the TTT, groups of CETA trainers-in-training (pairs, or triplet groups) will each facilitate separate technology-delivered CETA training to the new prospective CETA counselors from our partnering organizations. Trainers-in-training will facilitate technology-delivered trainings to prospective counselors, which include training on standard CETA and T-CETA delivery. Each training will be conducted with approximately 25 counselors (*N* = up to 50).

### Outcomes

The primary outcomes in the trial will be AYA trauma symptoms (as measured by the CPSS), internalizing symptoms, and externalizing behaviors (both measured by the YSR) administered via ACASI as described in the “Screening and baseline assessment” section. Secondary outcomes among AYA, also administered via ACASI, include substance use, which will be measured with the Alcohol, Smoking, and Substance Involvement Screening Test (ASSIST) [67, 77] and aggression/violence as measured by the Youth Victimization Scale [78]. Outcomes are assessed at baseline, post-treatment completion (approximately 3–4 months post-baseline for TAU participants), and 6 months post-treatment completion (approximately 9 months post-baseline for TAU participants). The primary time point is post-treatment.

In addition to AYA outcomes, we will collect preliminary data on counselor and trainer competency, fidelity, and knowledge. This will be collected at various points throughout the study through standardized role-plays and CETA knowledge tests. Mixed methods interviews will be conducted among clients, counselors, and trainers to explore the acceptability and feasibility of CETA training and delivery. We will also collect data on health utility among AYA using the EuroQol 5-Dimensions for Youth (EQ-5D-Y) [79, 80] to inform the cost-effectiveness analyses. In all, AYA participants will be engaged in the study for approximately 9–12 months.

### Data management

All data collected on paper forms will include only the participant’s ID number. Paper forms will be transferred securely in study vehicles to the storage site where they will be kept in locked filing cabinets within locked offices. Electronic data capture using the ACASI program will also only include participant ID numbers and be stored on encrypted drives.

### Data analysis

The analysis will be done using an intent-to-treat approach. Mixed effects regression models will be estimated for each mental/behavioral health outcome. Models will include fixed effects of treatment group, time, and a group × time interaction term. Random effects will include client ID and counselor ID. There will be two sets of analyses. First, we will conduct a superiority analysis, in which each of the CETA arms (CETA and T-CETA) is separately compared to TAU. Second, we will conduct a non-inferiority analysis in which we will compare the outcomes between the CETA and T-CETA groups.

The total number of AYA participants will be *N* = 400. We conducted power calculations for each of the three primary outcomes for which AYA will be enrolled in the study (trauma symptoms, internalizing symptoms, externalizing behaviors). All three required similar sample sizes; trauma symptoms required the largest sample size and are presented here. Based on a previous study with AYA using the same trauma symptom scale as this study (CPSS) [73], we hypothesize a baseline mean value of approximately 19.0 and a standard deviation of 13. For the superiority analyses, in which we will separately compare each CETA condition (in-person CETA and T-CETA) to the TAU control, we will assume an alpha = 0.025 to account for two comparisons. Further assuming power = .80 and an expected minimum effect size of each CETA condition compared to TAU of 0.5, we would require 78 persons per arm. For the non-inferiority analysis, in which we will compare the two CETA conditions to each other, we assume an alpha = 0.05, power = 80%, and a non-inferiority margin of 4.7, which is equivalent to one-third of the anticipated standard deviation. Previous studies have indicated that differences in the outcomes that are less than one-third of the standard deviation are considered not practically meaningful. Based on these assumptions, we would require 114 in each CETA arm for the non-inferiority comparison. Using the more conservative estimate for the CETA conditions (*N* = 114 each) and the estimate for the TAU (*N* = 78) yields a total sample size of *N* = 306. Assuming 20–25% attrition based on previous studies, we have inflated the final sample size to *N* = 400 (*N* = 100 TAU, *N* = 150 CETA, *N* = 150 T-CETA).

The incremental cost-effectiveness analysis will compare the cost-effectiveness of CETA and T-CETA relative to TAU. We will first derive quality-adjusted life years (QALYs) from health utility reported at each follow-up time point by AYA and then estimate the differences in the mean QALYs gained per treatment condition over study follow-up using the mixed effects models detailed above. We will then divide incremental QALYs gained under CETA and T-CETA by the incremental cost of each relative to TAU from the economic perspective of the healthcare provider (i.e., payer’s perspective) to estimate the primary cost-effectiveness outcome: incremental cost per QALY gained. Sensitivity analyses will be undertaken to examine the uncertainty around the cost-effectiveness estimates, and further interpreted using cost-effectiveness acceptability curves that reveal to decision-makers the probability of the intervention being cost-effective compared to the alternative, given different (implicit monetary) values placed on incremental improvements in the outcome measurement and QALYs. Cost-effectiveness acceptability curves will be based on bootstrapped regressions (to account for non-normally distributed data) of the study group upon net benefits, controlling for clusters.

### Data and safety monitoring

In addition to ethical review boards, there will be a data and safety monitoring board (DSMB) with researchers from both Zambia and the USA in relevant areas of expertise. Before study initiation, the DSMB will review and approve all study protocols detailing formal procedures for reporting and tracking all adverse reactions, following study progress, and identifying any need for premature termination of the protocol. No interim analyses will be conducted by the DSMB since the intervention is not considered harmful and to avoid erroneous conclusions by running multiple analyses during the study. All major study protocol changes will undergo ethical review and will be updated in the clinical trials registry.

#### Dissemination

Trial results will be presented to local partners in Zambia, including partner recruitment sites and other social service organizations offering services for AYA populations, via community meetings and project reports. The main study findings will be shared via social media channels of the implementing teams in the USA and Zambia. The results will also be presented at conferences and submitted for publication in relevant journals.

## Discussion

Scalable systems of care, such as those being tested in this trial, are needed to close the mental health treatment gap in LMIC, particularly for populations at-risk for poor outcomes and low treatment adherence, such as adolescents and young adults. The need for telephone-based mental health services in LMIC has become widely discussed during the COVID-19 pandemic. Significant barriers to scale-up of evidence-based treatments in LMIC include a lack of feasible, sustainable training for local lay providers, and client access to care. This will be the first CETA trial that is specifically designed to evaluate the treatment effectiveness among an adolescent population and the first to feature a technology-based training modality and a telehealth delivery. This study is a first step to build a system of technology-driven training and treatment provision to expand access to evidence-based mental healthcare in Zambia and other LMIC settings. The need for effective, remotely delivered interventions is particularly relevant in the COVID-19 era.

There are several notable limitations to this study. First, this trial has a risk of social desirability bias in AYA reporting of symptoms, particularly in the CETA treatment arms given their therapeutic relationship with counselors. To combat this risk, we are using a standardized ACASI assessment system to measure symptoms at all time points, administered by research assistants and not by the counselors themselves. Second, this trial is occurring across several urban sites in the capital city of Zambia, and our participants will not be representative of AYA in more rural areas of Zambia and across other rural LMIC settings. Additional research will be needed on the delivery of these interventions to rural populations where T-CETA may actually be more feasible and acceptable because of access to private space and the high costs of transportation to a clinic.

If the CETA technology training platform is deemed feasible and comparably effective to in-person training models, and T-CETA is found to be as effective as in-person CETA for AYA in Zambia, we aim to expand these technology-driven, cost-effective training and treatment delivery methods across several contexts in sub-Saharan Africa and other LMIC globally.

## Data Availability

The datasets analyzed during the current study and statistical code are available from the corresponding author on reasonable request, as is the full protocol.

## Abbreviations

ACASI: Audio computer-assisted self-interviewing system
ASSIST: Alcohol, Smoking, and Substance Involvement Screening Test
AYA: Adolescent and young adult
CEACs: Cost-effectiveness acceptability curves
CETA: Common Elements Treatment Approach
CMF: Client monitoring form
CPSS: Child PTSD Symptom Scale
DSMB: Data and safety monitoring board
EBT: Evidence-based treatments
EQ-5D-Y: EuroQol 5-Dimensions for Youth
FCV-19S: Fear of COVID-19 Scale
HIC: High-income countries
LMIC: Low- and middle-income countries
NGO: Non-governmental organization
QALYs: Quality-adjusted life years
RCT: Randomized controlled trial
T-CETA: Telehealth-adapted version of CETA
TAU: Treatment as usual
TTT: Train the trainer
YSR: Youth Self Report
